# New construction of an animal model for the orthotopic transplantation of an ovarian tumor

**DOI:** 10.1186/1757-2215-7-64

**Published:** 2014-06-11

**Authors:** Hui Zhang, Xinping Gao, Yongan Yang, Weiming Wang, Jin Liu, Yijuan Liang, Hongli Wu, Jinjin Qin, Kun Pan, Yifeng Wang, Junrong Shi, Youju Ma

**Affiliations:** 1 Affiliated Hospital Of HeBei University, Baoding City, HeBei Province, China; 2 The Third Hospital Of Baoding/Baoding Cancer Hospital, Baoding City, HeBei Province, China; 3 Department of Obstetrics and Gynecology, ZhuJiang Hospital of Southem Medical University, Guangzhou City, Guangdong Province, China

**Keywords:** Animal model, Orthotopic transplantation, Tumor, Ovarian cancer

## Abstract

A new technique has successfully established the non-obese diabetic/severely combined immunodeficiency (NOD/SCID) mouse model of ovarian cancer. Under 4% chloral hydrate (0.1 mL/g dose) anesthesia, female mice were inoculated with tumor-cell suspension. The expression rate of OVCAR3 to CA125 was assessed using flow cytometry. The inoculated site was hand palpated and the signs and symptoms related to tumor growth were observed with the naked eye. The allophycocyanin (APC) indirectly labeled mouse-antihuman CA125 and fluorescein isothiocyanate (FITC)-labeled anti-mouse MHC Class I molecule (H-2K^d^/H-2D^d^) were observed using a confocal laser scanning microscope. The animal model of ovarian cancer constructed using this method can more directly reflect the characteristics of cancer cells. It provides reliable experimental results and presents a technical platform for the research of ovarian cancer stem cells.

## Introduction

There are several methods by which to construct an animal model of epithelial ovarian cancer: Nude Mice Model [1] of a subcutaneous transplanted tumor, Model [2] of an intraperitoneal transplanted tumor, Nude Mice Model [3] of an omentum transplanted tumor, and Model [4] of an orthotopic transplanted or metastatic tumor. Orthotopic transplantation has the potential for broad application because it provides a microenvironment similar to that of the human body. The present orthotopic transplantation technique [5-9] is to first construct a Nude Mice Model of a subcutaneous transplanted tumor to supply the organism with cancer-cell strains, extract a small mass of the tumor tissue, open the ovarian capsule in the mouse under a dissecting microscope, and transplant the tissue mass into the ovarian parenchyma. This technique can only indirectly reflect the characteristics of ovarian cancer-cell strains. Moreover, it is a very difficult procedure and requires special equipment, such as a dissecting microscope; therefore, a new technique was used in our experiment: the cell suspension was injected directly into the ovarian parenchyma of the mouse and successfully established the NOD/SCID mouse model of ovarian cancer.

## Materials & methods

### Materials

Female mice (non-obese diabetes accompanied by severe combined immunodeficiency, NOD/SCID), aged 6–8 weeks, and weighing 16–20 g were bred under a specific pathogen-free (SPF) environment and purchased from the Experimental Animal Center at Sun Yat-sen University (YUE DONG Number of Certificate of Conformity: SCXK2004-0011). The mice were kept in a sterile laboratory at a constant temperature of 25 ± 2°C and at a constant humidity of 45–50% and were fed inside the laminar air flow rack. One week later, the mice received an intraperitoneal injection of etoposide (10 mg/kg, diluted to 200 mL with Hank’s balanced salt solution [HBSS]). At the same time, a small piece of an estrogen sustained-release tablet was inserted using a trocar into the hypoderm of the neck to lower the immunity of the mouse and shorten the period of neoplasia. Six days later, the mice were inoculated with the tumor cell suspension under 4% chloral hydrate (0.1 mL/g dose) anesthesia.

The cell strain of human ovarian cancer (OVCAR3) was purchased from the Affiliated Cancer Hospital of Sun Yat-sen University. A 15% fetal calf serum RPMI 1640 culture solution was cultured at 37°C in a 5% CO_2_ incubator. The RPMI 1640 culture medium, fetal calf serum, and pancreatic enzyme were purchased from Life Technologies (USA). Matrigel was purchased from BD (USA) and the CA125 antigen was purchased from Abcam (DN: ab1107, Cambridge, UK). Allophycocyanin (APC)-marked second antibody and fluorescein isothiocyanate(FITC)-marked anti-mouse major histocompatibility complex (MHC) class I antibody were purchased from eBioscience, Inc. (San Diego, CA, USA). All the other reagents were from domestic analytical reagents.

### Methods

The expression rate of OVCAR3 to CA125 was measured using flow cytometry. The OVCAR3 cells in the exponential phase were collected, 2 μL/10^6^ CA125 cells were added, and the solution was incubated for 1 hat 4°C. The cells were then washed 3 times in the serum-free HBSS, the second antibody was added and incubated at 4°C for 30 min, and the cells were washed again 3 times in serum-free HBSS and loaded into a flow cytometer.

**Figure 1:**
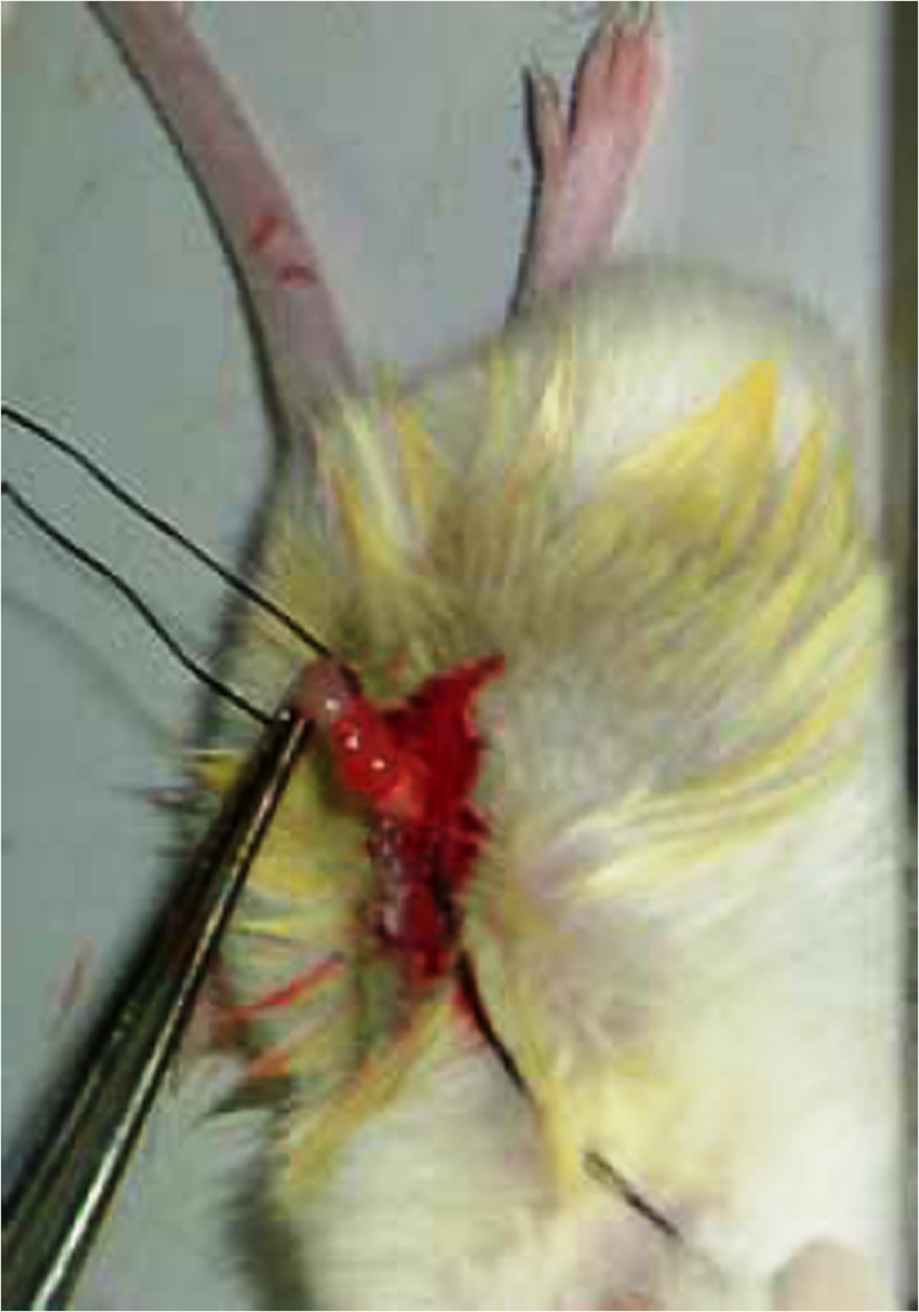
Data when mice were injected with cell suspension.

Routinely digest and collect cells in the exponential phase, living cells are counted and collected separately by centrifugation. When the density of the cells reached 5 × 10^7^/mL and 5 × 10^6^/mL, respectively, both of the solutions were suspended in 50 μL HBSS/Matrigel (1:1). The suction tube used for drawing up the Matrigel during the confecting period was precooled for 1 h at 4°C, then washed twice with 4°C HBSS. While the tube was being precooled and washed, the Matrigel was laid on the ice. The mice were randomly divided into groups A and B with 10 mice in each group. By injecting gentian violet into 110 mice (220 ovaries) having normal anatomical structures, it was noted that the maximum amount that could be injected into the mouse ovary was 20 μL; the mouse ovary would swell and break when >20 μL were injected. At 15 μL, the ovarian capsule was still intact; however, seepage was detected from the gentian violet injection site. The safe dose was determined to be 10 μL. At this level, the mouse ovarian capsule was observed after 10 min to be still intact and without any seepage. Each mouse was given an injection of 10 μL cell suspension in the right ovary, 1 × 10^5^/10 μL for group A and 2 × 10^4^/10 μL for group B. Following is a description of the procedure.With the mouse under complete anesthesia and in a prone position, the hair was removed from the area of the right subcostal and 1 cm from the vertebral column The area was sterilized with iodine alcohol, and a 1-cm incision was made parallel to the vertebral column using ophthalmic scissors The peritoneum was cut open and suspended with thread, the right ovary was exposed, the adipose tissue above the ovary was clipped with ophthalmic blunt-end forceps, and the uterus was clipped using another ophthalmic blunt-end forceps, making sure not to clip the ovarian capsule. The ovary was removed and, using the left hand, a 4-grade thread was threaded under the uterus (1.5 cm from the ovary) to suspend it. The right hand held the microinjector and used it to withdraw 10 μL cell suspension, and insert it into the uterine cavity, through the submucous membrane of the oviduct, and into the ovarian tissue. When the procedure was finished, the microinjector was removed (Figure 1) The thread suspending the uterus and the thread suspending the peritoneum were then removed, sprayed a few biomedical fibrin glue, and the uterus and ovary were placed back into the peritoneal cavity. Last, the peritoneum and the skin were sutured using a 7-grade absorbable surgical suture. The mice continued to be fed in an SPF environment after surgery.

Index observation: The inoculation site was palpated and the signs and symptoms related to tumor growth were observed, such as reduced subcutaneous fat, palpable masses in the abdomen, abdominal distention, a hump in the abdomen, and reduced activity. Each mouse was labeled based on the random table and each was weighed to obtain data to graph any weight changes. After approximately 50 days, there were signs and symptoms of reduced activity. After 3 months, the mice were anesthetized using the same protocols, both ovaries were removed, and the abdomen was opened to observe the status of tumor metastasis.

Pathological examination: Three months after inoculation, the inoculated ovary and the contralateral ovary were removed, fixed in 4% neutral formalin solution, embedded in paraffin, assume ordinary section and stained with hematoxylin and eosin as well as with immunohistochemical stain to detect the expression of CA125. The ordinary frozen sections were processed and labeled with fluorescent antibody. The APC indirectly labeled mouse-antihuman CA125 and FITC labeled anti-mouse MHC Class I molecule (H-2K^d^/H-2D^d^) were observed using a confocal laser scanning microscope.

### Ethical approval

The ethics committee is “河北大学医学伦理委员会” (Hebei University Medical Ethics Committee).

## Results

### Flow cytometer

**Figure 2:**
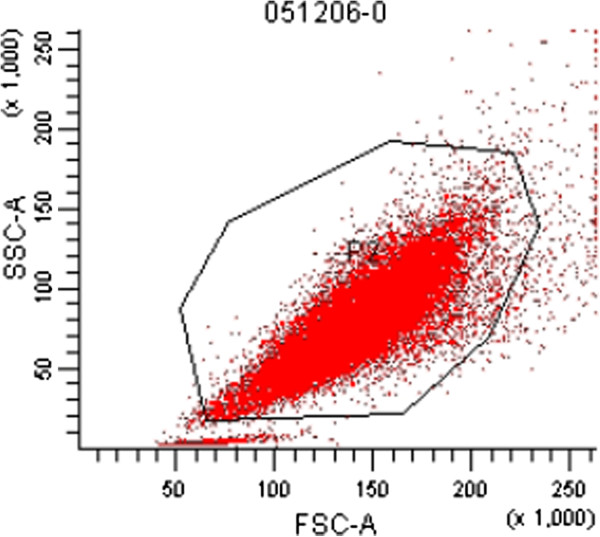
**More than 98% OVCAR3 expressed CA125.**

Flow cytometer testing results showed that >98% OVCAR3 cells expressed CA125 (Figure 2).

### Weight growth graph

**Figure 3:**
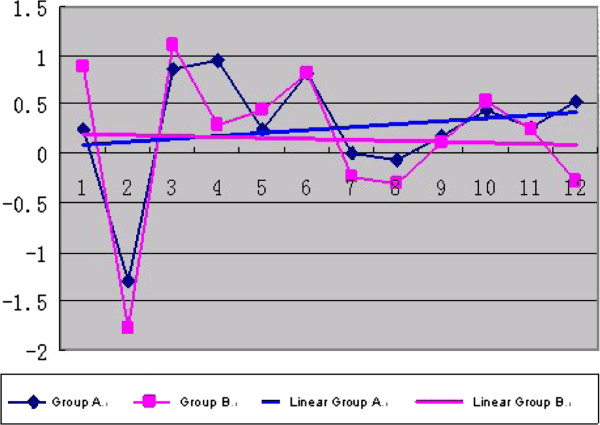
**Body weight decreased in the two groups of mice in the beginning as a result of the experimental pretreatment.** Linear A and Linear B group representing growth trend in body weight of mice in each group. Growth trend in body weight of mice in group B is less than that in group A.

A weight growth graph is shown in Figure 3. The line in the table indicates the growth trend.

### Morphological observation

**Figure 4:**
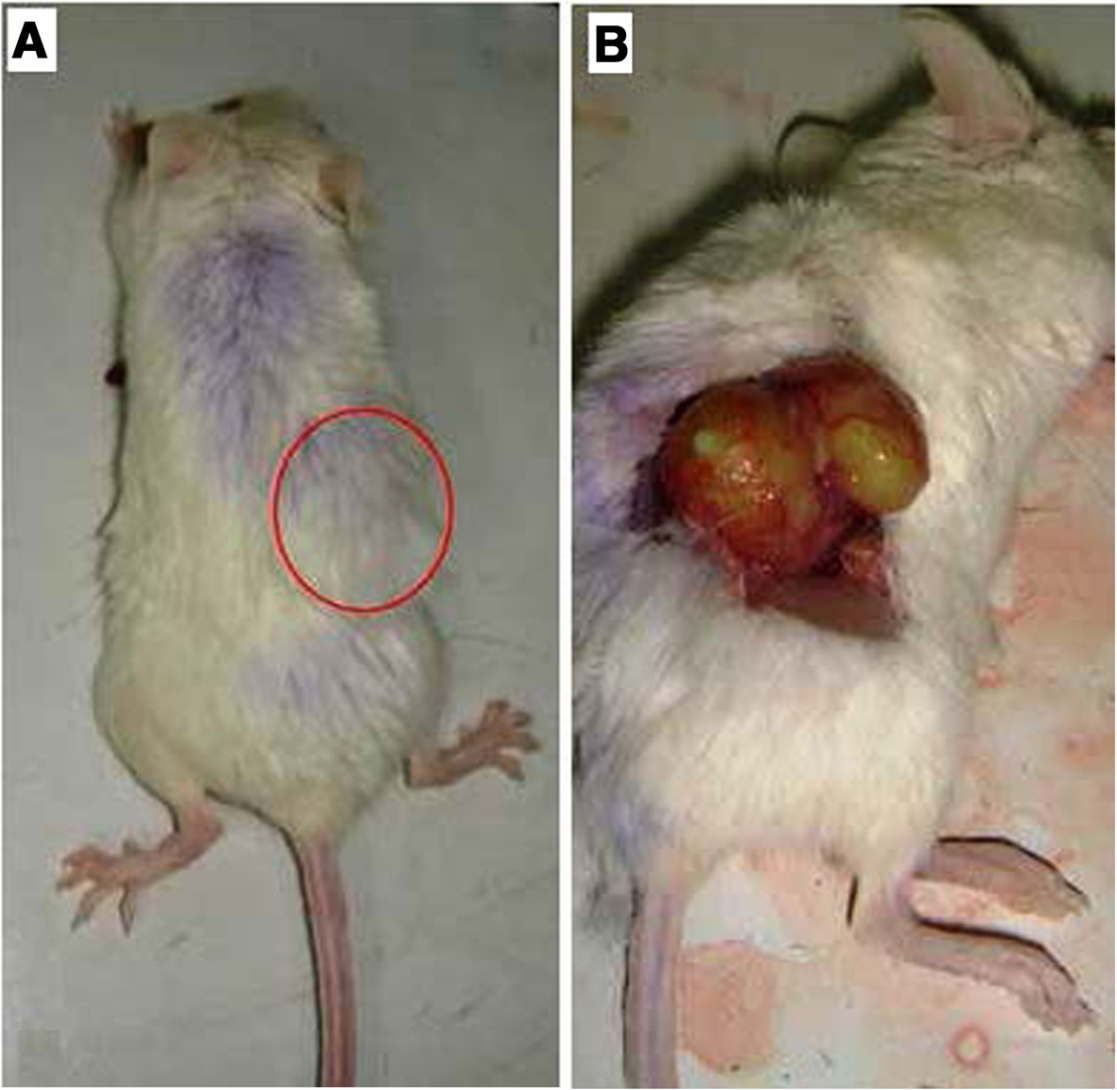
**A: Before laparotomy.** The injected right bottom side is higher than the surrounding skin. **B**: After laparotomy. Laparotomy can enable the observation of the planting of increased ovarian tumors.

**Figure 5:**
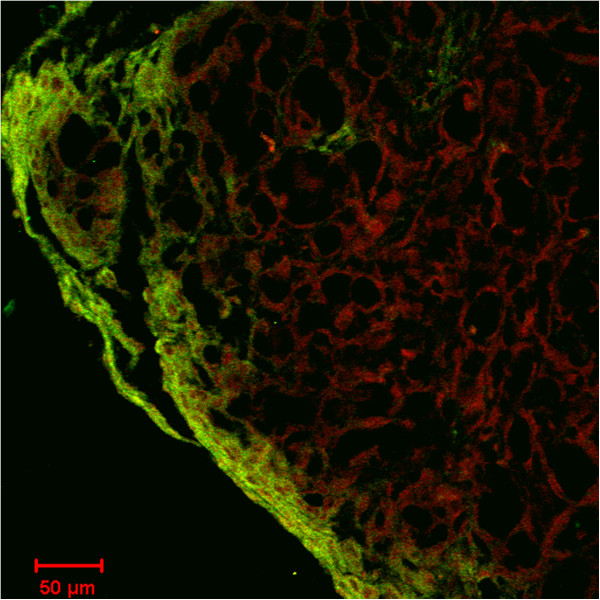
**Observation of ovary 3 months after inoculated OVCAR3 cells in group B under × 200 confocal laser scanning microscope.** Green light labels: ovary tissue of mouse. Red light labels: OVCAR3 transplanted tumor of human epithelial ovarian cancer.

**Figure 6:**
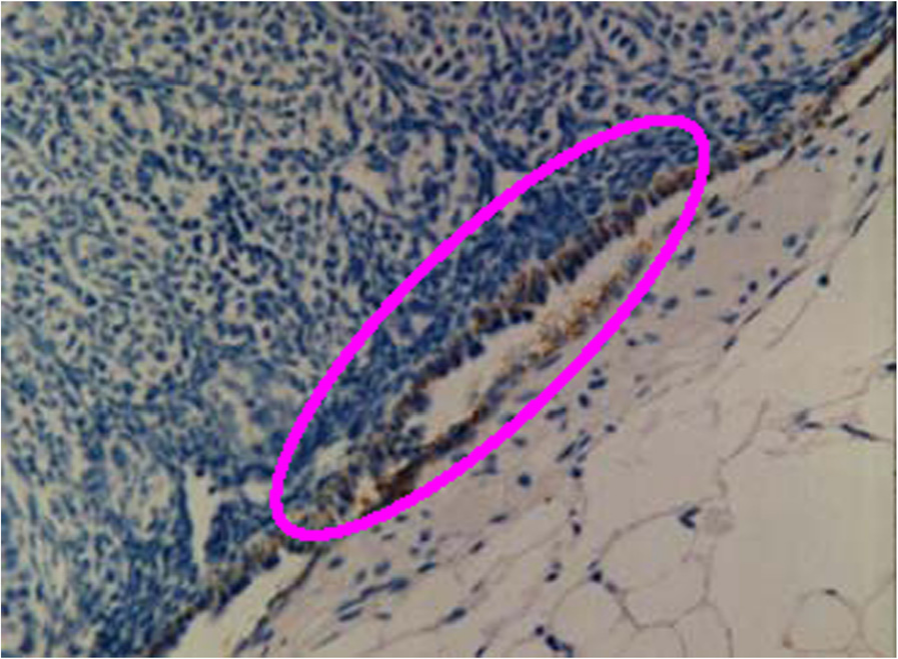
**Little amount of metastasis of ovarian cancer cells in contralateral ovary.** Immunohistochemical staining test CA125 × 200.

None of the 20 inoculated mice experienced broken ovarian capsules or seepage of the cell suspension. Only 1 microscopic neoplasia was observed in group A (1/10). All of the mice in group B (10/10) were observed as having ovarian neoplasia (Figure 4-A: before laparotomy, Figure 4-B: after laparotomy). The Pr values of both groups were measured using Fisher’s analysis (P < 0.05), and differences were observed between the 2 groups. The neoplasia cells in the 10 mice in group B were seen under the microscope in papillary arrangement. Secretions of papilla branches, glandular lumen in part of the regions, cubical or short columns of cells with wide trachychromatic irregular nuclei, and karyokinesis were observed, corresponding to the basic characteristics of OVCAR3 cells. In addition, the growth of ovarian cancer could be observed in the ovaries of the aforementioned 10 mice under a confocal laser scanning microscope. The FITC labeled anti-mouse MHC class I molecules (H-2K^d^/H-2D^d^) displayed green light labels in the mice cells, and the APC indirectly labeled mouse-antihuman CA125 displayed red light labels in the human epithelial ovarian cancer cells (Figure 5). In group B, 2 mice were found with contralateral ovarian metastasis (Figure 6), 1 was also found with 1- to 2-mL ascites and 1 with hepatic metastasis.

## Discussion

The T and B lymphocytes are absent in NOD/SCID mice. Processing with etoposide and an estrogen sustained-release tablet 6 days before transplantation decreased the immunity of the mice, thereby shortening the period of neoplasia. Mahammad et al. [10] successfully used this method in the construction of an animal model of mammary cancer and identified the markers of mammary cancer stem cells.

The characteristics of our method in constructing the model of orthotopic transplanted tumor. The animal model of ovarian cancer is an important method by which to study the biological behavior of ovarian cancer, to perform experimental treatments, and to select new and more effective treatments. 70 years ago, Biskind, G R. [11] removed the gonad of a mouse and implanted part or all of the removed ovary into the spleen. Under such conditions, the ovary remained active, but the gonadal hormones it generated were inactivated by the liver. The negative feedback mechanism resulted in an unbalanced gonadal hormone level in the mouse; therefore, neoplasia [11] was formed in the transplanted ovary. At a later stage19 years ago), Hilfrich [12] found that the cumulative effect of intravenous injection of dimethylbenzanthracene (DMBA) can accelerate neoplasia [12] of an ovary transplanted into the spleen. Such a method restricted the model’s broad application because of redundant experimental procedures and long observation periods. In recent years, many methods in the construction of such a model have been reported, such as subcutaneous, peritoneal, and orthotopic transplantation of tumors. Compared to subcutaneous or peritoneal transplanted tumor models, the orthotopic transplanted tumor model can better reflect the biological behavior of ovarian cancer. Because the ovary of a mouse is small, (0.5 cm in diameter), there has been no report of directly inoculating the cell suspension into the ovary. If this technique can be achieved, the animal model of human ovarian cancer will better simulate the occurrence and development of this type of cancer. We will be able to study not only the cell strain but also the biological behavior of the primary human ovarian cancer cells. The present method of constructing the orthotopic transplanted tumor model can only indirectly reflect the characteristics of the cell strains, and was restricted to processing tissue mass but not cell suspension. The difference between the previous orthotopic transplantation and our experiment is the direct inoculation of cell suspension into the mouse ovary. This method is the first of its kind. Because the ovary of the NOD/SCID mouse is comparatively small, the cancer cells are scattered. Microinjectors were used to pass through the *cornu uteri* and into the ovarian parenchyma to inject the cell suspension and to avoid the human-made cultivation of a tumor. In our experiment, 2 cell densities were set up: 1 × 10^5^/10 μL for group A and 2 × 10^4^/10 μL for group B. The results were that 1/10 in group A and 10/10 in group B were observed as having ovarian neoplasia. After analyzing the data, we found that the reason for such a result was that the ovary of the mouse is small, and that the high cell concentration might result in insufficient nutrients to the cells and, ultimately, cell death. Fisher reliable inspection results revealed that 2 × 10^4^/10 μL is the ideal inoculation concentration suitable for the size of the mouse ovary in this experiment.

Compared to previous methods, this method takes less time for the neoplasia period, assumes simple operational techniques, and can be performed under orthophoria conditions. Using this model, we are able to study not only the biological characteristics of cell strains, but the biological characteristics of primary ovarian cancer cells as well to further lay a foundation for the marker of ovarian cancer stem cells and provide an important tool for studying the biological behaviors of ovarian cancer, carrying out experimental treatments, and selecting more effective treatments.

The Matrigel used in the experiment is a type of protein extracted from ESH mouse sarcoma, which can produce basement membranes. It can adjust the biological behavior of epithelial cells, promote the increment and differentiation, etc. Some experiments [13] have proved that Matrigel can remarkably promote the formation and increment of an implant of uterine cancer, early and neat neoplasia, and rapid growth, and at the same time, keep the original differentiation, improve the utilization of animals, and shorten experimental periods.

The mouse MHC class I molecule (H-2K^d^/H-2D^d^) is expressed in nearly all of the mouse tissues, and >99% OVCAR3 expressed CA125 through flow cytometry. The results of detecting neoplasia in the ovary of a NOD/SCID mouse with these 2 markers are objective and reliable.

## Conclusion

The animal model of ovarian cancer constructed under this method can reflect the characteristics of cancer cells even more directly than previous models. This model takes less time for neoplasia, assumes simple techniques, provides reliable experimental results, and presents a technical platform for research in ovarian cancer stem cells. It is worth additional research in the construction of the orthotopic transplanted tumor model of ovarian cancer.

### Novelty and impact statements

A suspension of ovarian cancer cells were microinjected directly into a mouse ovary to observe their tumorigenicity. This model is a real sense of the orthotopic transplantation tumor model. With this model, we can study the biological characteristics of ovarian tumor cells, such as drug resistance and the biological markers for cancer stem cells.

## Competing interests

We certify that there is no conflict of interest with any financial organization regarding the material discussed in the manuscript. All authors have participated sufficiently in this work to take public responsibility for it. All authors have reviewed the final version of the manuscript and approved it for publication. Neither this manuscript nor one with substantially similar content under my (our) authorship has been published or is being considered for publication elsewhere; this manuscript has been submitted with the full knowledge and approval of the institution or organization given as the affiliation of the author.

## Authors’ contribution

HZ is responsible for the experimental operation and writing papers; XG is responsible for feeding the mice; YY is responsible for recording the relevant experimental data do statistical analysis; WW, JL, YH, JK are responsible for pathological examination, fluorescence microscopy, YW is responsible for financial support. In addition, JS and YM are responsible for experimental guidance. SJS (Email: 18903363120@163.com) is added to author 11 and YM (Email: 18903363120@163.com) is added to author 12. All authors read and approved the final manuscript.
